# Prevalence and its risk factors for low back pain among operation and maintenance personnel in wind farms

**DOI:** 10.1186/s12891-016-1180-y

**Published:** 2016-07-26

**Authors:** Ning Jia, Tao Li, Shuangqiu Hu, Xinhe Zhu, Kang Sun, Long Yi, Qiong Zhang, Guilian Luo, Yuzhen Li, Xueyan Zhang, Yongen Gu, Zhongxu Wang

**Affiliations:** 1 Department of Occupational Protection and Ergonomics, National Institute of Occupational Health and Poison Control, Chinese Center for Disease Control and Prevention, NO.29, Nanwei Road, Xicheng District, Beijing, 100050 People’s Republic of China; 2 Labor Health Occupational Disease Prevention and Control Center in Zhuzhou, Zhuzhou, 412011 Hunan People’s Republic of China; 3 Wind power Division of Zhuzhou Electric Locomotive Institute Corporation, China South Locomotive and Rolling Stock (CSR), Zhuzhou, 412007 Hunan People’s Republic of China; 4 Hunan University of Technology, Zhuzhou, 412007 Hunan People’s Republic of China

**Keywords:** Wind farms, Low back pain, Risk factors, Ergonomic

## Abstract

**Background:**

With the increasingly severe energy shortage and climate change problems, developing wind power has become a key energy development strategy and an inevitable choice to protect the ecological environment worldwide. The purpose of this study was to investigate the prevalence of low back pain (LBP) and analyze its risk factors among operation and maintenance personnel in wind farms (OMPWF).

**Methods:**

A cross-sectional survey of 151 OMPWF was performed, and a comprehensive questionnaire, which was modified and combined from Nordic Musculoskeletal Questionnaires (NMQ), Washington State Ergonomics Tool (WSET) and Syndrome Checklist-90(SCL-90) was used to assess the prevalence and risk factors of LBP among OMPWF.

**Results:**

The prevalence of LBP was 88.74 % (134/151) among OMPWF. The multivariable model highlighted four related factors: backrest, somatization, squatting and lifting objects weighing more than 10 lb more than twice per minute.

**Conclusions:**

The prevalence of LBP among OMPWF appears to be high and highlights a major occupational health concern.

## Background

With the increasing scarcity of the world’s energy, wind energy is viewed as a low-carbon, clean, and abundant source of renewable energy, which is especially popular and has become an important measure to improve energy structure, reduce environmental pollution, and protect the ecological environment all over the world. However, the wind farm industry may also lead to serious health threats to operation and maintenance personnel in wind farms (OMPWF) whilst bringing many benefits in energy conservation.

Regularly OMPWF need to carry out the inspection, maintenance, and fault solutions of various equipment in wind turbine nacelle. They are required to climb the wind turbine tower several times every day, which is up to about 80 m high from the ground, causing great physical exertion. Since the wind turbine nacelle is narrow and small, and almost all operation activity is manual, workers are forced to spend long periods of time in awkward postures. This may lead to many adverse ergonomic issues, such as heavy physical labor, repetitive tasks, lifting and excessive force. In addition, wind farms are generally built in remote areas with abundant wind energy resources, which include the ridge, grassland, Gobi Desert, and island, etc. Enterprises have a regulation of holidays by rotational schedule, that is, OMPWF would go home to rest after work in wind farms for 2-3 months continuously. The environment of their resident is relatively isolated, less time for recreation, and a long time of being away from family and friends. All these factors lead to social isolation which is harmful for OMPWF’s psychosocial health.

Preliminary investigations and related research have confirmed that adverse ergonomic, psychosocial, and individual and lifestyle factors mentioned above might be associated with the risk of low back pain (LBP) [1, 2]. LBP is the most common musculoskeletal disorders, which not only seriously influences the health, working capacity and professional life of workers, but also brings heavy burden to their families and society. LBP has been included in the list of compensation diseases in many industrialized countries. It is estimated that LBP has resulted in a loss of 149 million working days and has caused direct and indirect economic losses of up to one hundred to two hundred billion dollars [3]. It has cost Germany more than 7,000 Euros annually owing to LBP [4]. The global burden of disease research showed that workplace adverse ergonomics caused by LBP gave rise to 21.7 million disability-adjusted life years [5].

So far, there are few studies on the occupational health issues caused by new energy industries despite programs of clean energy developing quickly in China. The purpose of this study was to explore the occurrence of LBP and identify the risk factors of LBP among OMPWF. The occurrence of LBP influenced by adverse ergonomic issues, psychosocial problems, and lifestyle factors are discuss in this study, which provided useful information for strategies and measures to prevent and reduce the occurrence of LBP, therefore offering scientific basis for healthy and sustainable development of the clean energy industry.

## Methods

### Subjects

A questionnaire based cross-sectional study was carried out among OMPWF in a wind turbine manufacturing enterprise in China, which involved 17 wind farms. Subjects eligibility criteria were as follows: male, having worked no less than 1 year in the current position, no history of significant trauma, no diagnosed rheumatic or tumour, and having never had an accident involving the low back region previously. All subjects who met the eligibility criteria were selected.

### The workspace of the nacelle

OMPWF’s routine work is mainly conducted in nacelle, which is roughly 4 m high, 10 m long, and 4 m wide. There are large-scale instruments, such as generator, gearbox, battery cupboard, and yaw control system located in the middle of the nacelle, which occupy the most of the interior space in nacelle. The maintenance passageway is approximately 0.8 m wide. Since all of these make the working space very narrow, OMPWF have to adopt adverse postures, such as stoop, squat, and prone position. The space of hands operation is only up to 0.1 m wide when overhauling the generator. The operating point from the nacelle wall is only 0.6 m wide when maintaining the gearbox. The battery cupboard from the nacelle wall is only 0.7 m wide, when replacing batteries in the battery cupboard. OMPWF are compelled to lie on the gearbox anointing with oil for yaw gear ring when maintaining the yaw control system, the distance between yaw gear ring and the lay flat is only 0.3 m wide.

### Questionnaire

In this study, the data were obtained with a comprehensive questionnaire based on Nordic Musculoskeletal Questionnaire (NMQ) [6], Washington State Ergonomics Tool (WSET) [7], and Symptom Checklist 90(SCL-90) [8] and combined with the actual situation of the operation maintenance operation in wind farms.

#### Consequences of low back pain

In our study, the diagnostic procedures of LBP included questionnaires and palpation inspection. First, patients were selected by questionnaire in which they complained of any two kinds of symptoms of ache, numbness, pain, or discomfort in the low back simultaneously and which could not be relieved after 24 h of rest. Additionally, LBP-positive patients would be further diagnosed by orthopedic surgeons through palpation inspection on those complaining of LBP.

A modified version of the Nordic Musculoskeletal Questionnaire(NMQ) was used to assess the cumulative pain prevalence in the low back in the past 12 months. The validity and reliability of the NMQ has been validated in previous studies [9], and this questionnaire has later been translated into Chinese [10]. The NMQ has three sections. The first section covered demographic characteristics such as age, job tenure, height, weight, education, tobacco smoking, and alcohol consumption. The second section recorded whether operators had experienced ache, pain, or discomfort in their low back in the past 12 months. The third section of the questionnaire included items about living environment, habits: the height of desk/chair, space below the table, height of keyboard/ mouse, height of the backrest, and so on.

#### Ergonomic, psychosocial risk factors assessments

Ergonomic risk factors were assessed through Washington State Ergonomics Tool (WSET). The WSET uses observational checklist methodology to evaluate generic risk factors in the following six major categories: awkward posture, highly repetitive motion, high hand force, repeated impact, lifting, and hand-arm vibration. Employers could use this tool to determine whether the job activity increased the risk of employees’ low back pain.

The Chinese version of Symptom Checklist-90 (SCL-90) is a widely-used self-report symptom inventory that consists of 90 items. This version is used to assess psychosocial distress symptoms among patients with LBP during the preceding week. Multiple studies have found that the Chinese version of the SCL-90 has satisfactory reliability and validity [11]. Moreover, the following subscales are derived from the 90 items: somatization, obsessive-compulsive, interpersonal-sensitivity, depression, anxiety, hostility, phobic-anxiety, paranoid ideation, and psychoticism. Each symptom is rated on a 5-point Likert scale (0 = notatall,4 = extremely) indicating how frequently the client has experienced these symptoms in the last week. The total score is inversely related to the psychological health status, the higher the total score, the worse the psychological health status.

### Data analysis

Analysis of the data was performed with IBM SPSS software version 20. Descriptive statistics were conducted for demographic characteristics, psychosocial distress status, and LBP prevalence rates. Chi-square test was used to determine differences between categorical variables. The stepwise logistic regression was used to identify the associations between the ergonomic, psychosocial, and other related factors possibly related to low back pain. The associations were estimated by calculating the ORs and their 95 % CI. *P*-value thresholds for entry to and removal from the multivariate model was set at *P* <0.05 and 0.10 respectively.

## Results

### Study population characteristics

The questionnaires were completed by 151 male OMPWF. The overall response rate was 100 %. Demographic characteristics of the participants are shown in Table 1. In total, the mean age was 25.96 years, and the mean working hours per week were 44.70 h. The average height and weight was 171.89 ± 5.41 cm and 66.87 ± 9.26 kg, respectively. The average working-age was 3.60 ± 2.19 years. Educational level of the participants was high, as 99.34 % of the participants had at least a Bachelor’s degree.

**Table 1:** Demographic characteristics of the participants (*n* = 151)

Characteristics		N (%)	Mean (SD)
Age (yr)	<25	48 (31.79)	25.96 (2.26)
	25~	90 (59.60)	
	30~	13 (8.61)	
Height (cm)	<170	53 (35.10)	171.89 (5.41)
	170~	49 (32.45)	
	175~	49 (32.45)	
Weight (kg)	<60	31 (20.53)	66.87 (9.26)
	60~	60 (39.74)	
	70~	60 (39.74)	
Working-age (yr)	<1	27 (17.88)	3.60 (2.19)
	1~	22 (14.57)	
	2~	102 (67.55)	
Working hours per week (h)	<40	13 (8.61)	44.70 (1.86)
	40~	79 (52.32)	
	45~	59 (39.07)	
Education	High school	1 (0.66)	
	Bachelor’s degree	136 (90.07)	
	Higher than Bachelor’s degree	14 (9.27)	

### Prevalence of LBP

According to questionnaire and palpation, the prevalence of LBP was 88.74 % (134/151).

### Adverse ergonomic factors exposures

Table 2 shows the association between adverse ergonomic factors and the prevalence of LBP in the univariate analysis. LBP was significantly associated with awkward posture (squatting more than 4 h total per day) (OR = 8.80, 95 % CI1.15–67.10, *P* < 0.05); heavy, frequent, or awkward lifting (lifting objects weighing more than 10 lb if done more than twice per minute, more than 2 h total per day) (OR = 3.77, 95 % CI1.29–11.01, *P* < 0.05); repeated impact (using the knee as a hammer more than once per minute, more than 2 h total per day) (OR = 2.83, 95 % CI1.01–7.92, *P* < 0.05); high hand force (gripping an unsupported object(s) weighing 10 lbs or more per hand, or gripping with a force of 10lbs or more per hand, meanwhile no other risk factors more than 4 h total per day) (OR = 1.14, 95 % CI1.07–1.21, *P* < 0.05).

**Table 2:** Adverse ergonomic factors of LBP among OMPWF with univariate analysis

Adverse ergonomic risk factors	Low back pain			
	Number of workers	Case	OR	(95 % CI)
Awkward posture				
Working with the hand(s) above the head, or the elbows above the shoulders, more than 4 h total per day				
No	6	4	1	
Yes	145	130	4.33	(0.73–25.68)
Repeatedly raising the hand(s) above the head, or the elbow(s) above the shouder(s) more than once per minute, more than 4 h total per day				
No	12	10	1	
Yes	139	124	1.65	(0.33–8.27)
Working with the neck bent more than 45° (without support or the ability to vary posture), more than 4 h total per day				
No	14	11	1	
Yes	137	123	2.40	(0.60–9.63)
Working with the back bent forward more than 30° (without support or the ability to vary posture), more than 4 h total per day				
No	10	8	1	
Yes	141	126	2.10	(0.41–10.82)
Working with the back bent forward more than 45° (without support or the ability to vary posture), more than 2 h total per day	21	17	1	
No	130	117	2.12	(0.620–7.25)
Yes				
Squatting more than 4 h total per day				
No	4	2	1	
Yes	147	132	8.80	(1.15–67.10)^*^
Kneeling more than 4 h total per day				
No	32	27	1	
Yes	119	107	1.65	(0.54–5.09)
High hand force				
Pinching an unsupported object(s) weighing 2 lbs or more per hand, or pinching with a force of 4 lbs or more per hand, meanwhile highly repetitive motion more than 3 h total per day				
No	22	10	1	
Yes	129	114	0.76	(0.16–3.58)
Pinching an unsupported object(s) weighing 2 lbs or more per hand, or pinching with a force of 4 lbs or more per hand, meanwhile hand/wrist in awkward posture more than 3 h total per day				
No	28	23	1	
Yes	123	111	2.01	(0.65–6.26)
Pinching an unsupported object(s) weighing 2 lbs or more per hand, or pinching with a force of 4 lbs or more per hand, meanwhile no other risk factors more than 4 h total per day				
No	22	18	1	
Yes	129	116	1.98	(0.58–6.76)
Gripping an unsupported object(s) weighing 10lbs or more per hand, or gripping with a force of 10 lbs or more per hand, meanwhile highly repetitive motion more than 3 h total per day				
No	15	12	1	
Yes	136	122	2.179	(0.55–8.67)
Gripping an unsupported object(s) weighing 10lbs or more per hand, or gripping with a force of 10 lbs or more per hand, meanwhile hand/wrist in awkward posture more than 3 h total per day				
No	27	23	1	
Yes	124	111	1.49	(0.44–4.97)
Gripping an unsupported object(s) weighing 10lbs or more per hand, or gripping with a force of 10 lbs or more per hand, meanwhile no other risk factors more than 4 h total per day				
No	10	10	1	
Yes	141	124	1.14	(1.07–1.21)^*^
Highly repetitive motion				
Using the same motion with little or no variation every few seconds, and high, forceful exertions with the hand(s) more than 2 h total per day				
No	28	25	1	
Yes	123	109	0.93	(0.25–3.50)
Using the same motion with little or no variation every few seconds, meanwhile no other risk factors more than 6 h total per day				
No	32	29	1	
Yes	119	105	0.78	(0.21–2.88)
Intensive keying and hand / wrist in awkward posture more than 4 h total per day				
No	26	24	1	
Yes	125	110	0.61	(0.13–2.85)
Intensive keying and no other risk factors more than 7 h total per day				
No	31	29	1	
Yes	120	105	0.44	(0.10–2.23)
Repeated impact				
Using the hand (heel/base of palm) as a hammer more than once per minute, more than 2 h total per day				
No	41	34	1	
Yes	110	100	2.06	(0.73–5.83)
Using the knee as a hammer more than once per minute, or more than 2 h total per day				
No	55	45	1	
Yes	96	89	2.83	(1.01–7.92)^*^
Heavy, Frequent or Awkward Lifting				
Lifting object weighing more than 75 lb and more than 10 times per day				
No	115	104	1	
Yes	36	30	0.53	(0.18–1.55)
Lifting object weighing more than 55 lb and more than 10 times per day				
No	13	11	1	
Yes	138	123	1.49	(0.30–7.38)
Lifting objects weighing more than 10 lb if done more than twice per minute, or more than 2 h total per day				
No	28	21	1	
Yes	123	113	3.77	(1.29–11.01)^*^
Lifting objects weighing more than 25 lb above the shoulders, below the knees or at arms length more than 25 times per day				
No	27	23	1	
Yes	124	111	1.49	(0.44–4.97)
Moderate to high hand-arm vibration				
Using impact wrenches, carpet strippers, chain saws, percussive tools (jack hammers, scalers, riveting or chipping hammers) or other tools that typically have high vibration levels, more than 30 min total per day				
No	52	44	1	
Yes	99	90	1.82	(0.66–5.03)
Using grinders, sanders, jigsaws or other hand tools that typically have moderate vibration levels more than 2 h total per day				
No	46	41		1
Yes	105	93	0.95	(0.31–2.86)

*LBP* low back pain, *OR* odds ratio, *CI* confidence interval

*Significant at *p* < 0.05

### Psychosocial risk factors exposures

Table 3 shows the means and standard deviation on the subscales of the SCL-90 in the LBP positive group were significantly higher than those in negative group (*P* < 0.05). The three items with highest scores were obsessive compulsive, somatization, and depression.

**Table 3:** Comparison of psychosocial health situations in the LBP positive and negative groups among OMPWF

Subscale	The LBP positive groups(*n* = 134)		The LBP negative groups(*n* = 17)		*t*	*P*
	Mean	S.D.	Mean	S.D.		
Somatization	1.77	0.59	1.36	0.37	8.115	0.000
Obsessive compulsive	1.83	0.59	1.56	0.51	4.405	0.000
Interpersonal sensitivity	1.57	0.62	1.39	0.41	3.404	0.001
Depression	1.64	0.61	1.46	0.44	3.463	0.001
Anxiety	1.53	0.58	1.38	0.45	3.028	0.003
Hostility	1.51	0.58	1.34	0.38	3.390	0.001
Phobic anxiety	1.27	0.45	1.17	0.28	2.433	0.016
Paranoid ideation	1.52	0.58	1.38	0.38	2.812	0.006
Psychoticism	1.52	0.53	1.29	0.36	5.079	0.000

*LBP*, low back pain

### Body dimensions factors exposures

**Fig. 1:**
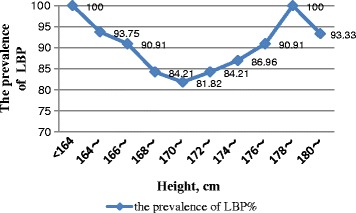
The prevalence trends of LBP by different height levels

**Fig. 2:**
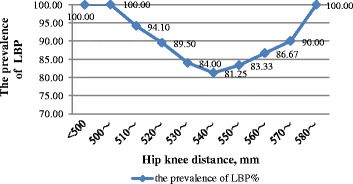
The prevalence trends of LBP by different hip knee distance levels

Figures 1 and 2 show the prevalence of LBP among participants with height less than 168 cm or higher than 176 cm was significantly higher than those with height ranging from168 to 176 cm, presenting a concave characteristic. Although the sample size of this study was small, there was a changing trend of concave, namely, the prevalence of LBP among those whose hip knee distance was less than 510 cm or longer than 570 cm was significantly higher than those with hip knee distance ranging from 510 to 570 cm.

### Individual and lifestyle factors exposures

The individual and lifestyle factors are presented in Table 4. There was a significant association between LBP and some individual and lifestyle factors (i.e. using computer during your spare time and the height of desk).

**Table 4:** Individual and lifestyle factors of LBP among OMPWF with univariate analysis

Variable	Number of workers	Cases	LBP (%)	χ^2^	*P*
Age					
< 25	48	41	85.4	0.839	0.657
25~	90	81	90.0		
30~	13	12	92.3		
Working-age					
< 1	27	24	88.9	0.194	0.907
1~	64	56	87.5		
3~	60	54	90.0		
Weight (kg)					
< 60	31	27	87.1	0.856	0.652
60~	60	55	91.7		
70~	60	52	86.7		
Height (cm)					
< 170	53	48	90.6	3.949	0.139
170~	49	40	81.6		
175~	49	46	93.9		
Physical exercise					
yes	38	34	89.5	0.027	1.000
no	113	100	88.5		
Smoking					
Yes	57	51	89.5	0.049	1.000
No	94	83	88.3		
Drinking					
Yes	130	114	87.7	1.030	0.469
No	21	20	95.2		
Using the computer time during your spare time(h)					
< 1	17	17	100	11.926	0.008
1~	32	26	81.2		
2~	51	50	98.0		
3~	51	41	80.4		
The height of chair(cm)					
< 35	17	15	88.2	0.086	0.958
> 50	21	19	90.5		
35–50	111	98	88.3		
The height of desk (cm)					
< 50	76	68	89.5	9.691	0.008
> 72	50	48	96.0		
50–72	25	18	72.0		
Legroom underneath the desk					
Yes	111	96	86.5	1.906	0.240
No	38	36	94.7		
Whether the keyboard and mouse at the same height					
Yes	137	120	87.6	1.681	0.362
No	12	12	100.0		
Backrest					
Yes	45	36	80.0	3.610	0.069
No	46	43	93.5		

*LBP* low back pain

### Multivariable model predicting LBP

The risk factors of LBP among OMPWF predicted by multivariable logistic regression model are shown in Table 5. The multivariable model showed that after adjusting for other factors, squatting more than 4 h total per day (adjusted odds ratio (AOR) 3.10, 95 % CI 1.10 to 8.80), lifting objects weighing more than 10 lb more than twice per minute, more than 2 h in total per day (AOR 4.29, 95 % CI 1.15 to 15.94), and somatization (AOR 2.70, 95 % CI 1.48 to 4.91) were positively associated with LBP, while backrest was inversely associated with LBP(AOR 0.36, 95 % CI 0.20 to 0.67).

**Table 5:** Multivariate logistic regression model predicting the risk factors of LBP among OMPWF

Variable	Coefficient	Waldχ^2^	AOR	95%CI	*P* value
Backrest	−1.010	10.800	0.36	0.20–0.67	0.001
Somatization	0.992	10.595	2.70	1.48–4.91	0.001
Squatting more than 4 h total per day	1.133	4.543	3.10	1.10–8.80	0.033
Lifting objects weighing more than 10 lb if done more than twice per minute, more than 2 h total per day	1.455	4.718	4.29	1.15–15.94	0.030

*AOR* adjusted odds ratio, *CI* confidence interval

## Discussion

There have been a large number of available data on the prevalence of the LBP in traditional industries, such as manufacturing [12], automotive industry [13], health care industry [14], and steel industry [15], while there is little information about these issues in the wind power industry. This study revealed the prevalence of LBP on the OMPWF was up to 88.74 % in the past 12 months. This is higher than the yearly prevalence of LBP reported on other occupational group in the literature, which varies from 20 to 68 % [16–19]. These findings suggest that OMPWF are at high-risk of suffering from LBP.

In this study, multivariable logistic regression analysis revealed a number of correlates of LBP including adverse ergonomic, psychosocial, or lifestyle factors. Of the adverse ergonomic factors, OMPWF who reported squatting more than 4 h per day were 3.10 times more likely to suffer from LBP than those who did not. Our study supports previous findings highlighting the prolonged static postures, particularly the squatting position as the most aggravating factor to be associated to LBP [20]. In addition, the strongest association in this cross-sectional survey was observed between LBP and lifting objects weighing more than 10 lb more than twice per minute for more than 2 h in total per day. In our study, heavy and awkward lifting was related to LBP with a high odds ratio of 4.29. This finding is in accordance with other research in which manual handling has previously been shown to be a common LBP risk factor. In Australia and New Zealand, manual handling in the preceding 12 months increased the likelihood of LBP among nurses and midwives [21]. Similarly, Okunribidofound manual handling increases risk for LBP among city bus drivers [22]. According to this investigation, OMPWF maintain and troubleshoot various engineering mechanical parts in the nacelle, which is a narrow and confined space for a long time. Due to the constraints of the dimension in the nacelle, OMPWF have been forced to maintain poor posture, which includes squatting, stooping, and using a straight ladder to climb. As reported previously, awkward posture was to be associated with LBP [23]. This is consistent with the findings of our study.

The present study indicated that the SCL-90 scores of LBP-positive group were higher than that of LBP-negative group, which means that mental health of the former was worse than the latter. In the subscales score of SCL-90, the obsessive compulsive score is highest followed by somatization and depression. It seems that the adverse psychosocial health among OMPWF was associated with LBP. In several studies, psychosocial factors, such as high job strain, high job dissatisfaction, obsessive compulsive, somatization, and depression have been reported to increase the LBP prevalence [1, 24, 25]. A 3-year follow-up study of the general working population in Norway showed that psychosocial factors appeared as the most consistent and important predictors of LBP [26]. In a study, Urquhart DM found a strong association between somatization and the prevalence of LBP [27]. From the view of physiology, adrenaline will be released and meanwhile blood flux accelerated when people become nervous or scared, resulting in motivated muscle activity to cope with stress [28]. However, the present study was cross-sectional in design, therefore it cannot provide any confirmatory evidence in favour of a cause-effect relationship between these two variables.

Despite the small sample size of this study, trends in the occurrence of in correlation with the body dimensions of an individual can be seen by our data. It is interesting to note that Figs. 1 and 2 show a “U-shaped” relationship between body dimensions and the prevalence of LBP. The height cut-points indicate that individuals with height less than 168 cm or higher than 176 cm have an increased risk. Therefore, it seems that the space of nacelle is more suitable for workers with a height between 168 and 176 cm in terms of ergonomics. Given that the proper range of body size is too narrow, wind turbine design engineers should consider redesigning the inner structure in nacelle based on the ergonomics to reduce the risk of LBP.

Our study indicate that using the computer during spare time and the height of desk could also influence LBP prevalence. According to OSHA ergonomic solutions, height-adjustable desks should generally be between 20 and 28 in. (50–72 cm) high [29]. This study also confirmed that the prevalence of LBP was at the lowest level within this height range. Furthermore, the multivariable logistic regression model showed that backrest was a protective factor. Thus, it might be indicated that from an ergonomic point of view, the most basic concepts of supporting the back in order to avoid bending more than 30° have been demonstrated to reduce the occurrence of the LBP [30].

In this study, LBP was diagnosed through self-reported questionnaire in combination with rigorous palpation inspection which might lower the recall bias. Nonetheless, the study still had several limitations. First, since the present study was cross-sectional, we could not establish causal inference. In future studies, longitudinal cohort studies should be more appropriate to further elucidate the causal correlates between those factors and the LBP consequences. Second, this study used only a small sample size of OMPWF in a large wind turbine manufacturer in China, which may not represent the industry-wide working conditions of operation and maintenance personnel in wind farms. Therefore, further studies with larger sample size are needed to improve the industry representation.

## Conclusions

It can be concluded that LBP appears to be a serious problem among OMPWF and highlights a major health concern. The association between some risk factors, such as adverse ergonomic factors (squatting more than 4 h total per day and lifting objects weighing more than 10 lb if done more than twice per minute, more than 2 h total per day), psychosocial factors (somatization), and individual, lifestyle factors (using the computer too long during spare time) and LBP were highlighted in this study. It is obviously essential to make intervention strategies concentrating on ergonomic factors (improving the narrow working space in the wind farms, reducing awkward or tiring positions) as well as the psychosocial factors (managing work stress, carrying out various forms of cultural and sports activities and psychological counseling and persuasion) to prevent and minimize the occurrence of LBP among OMPWF.

## Abbreviations

AOR, adjusted odds ratio; LBP, low back pain; NMQ, Nordic Musculoskeletal Questionnaires; OMPWF, operation and maintenance personnel in wind farms; SCL-90, syndrome checklist-90; WSET, Washington State Ergonomics Tool
